# Phylogeny and Taxonomy on Cryptic Species of Forked Ferns of Asia

**DOI:** 10.3389/fpls.2021.748562

**Published:** 2021-12-17

**Authors:** Zuoying Wei, Zengqiang Xia, Jiangping Shu, Hui Shang, Stephen J. Maxwell, Lijun Chen, Xile Zhou, Wang Xi, Bayu Adjie, Quan Yuan, Jianguo Cao, Yuehong Yan

**Affiliations:** ^1^Key Laboratory of National Forestry and Grassland Administration for Orchid Conservation and Utilization, The National Orchid Conservation Center of China and The Orchid Conservation and Research Center of Shenzhen, Shenzhen, China; ^2^Eastern China Conservation Centre for Wild Endangered Plant Resources, Shanghai Chenshan Botanical Garden, Shanghai, China; ^3^College of Life Sciences, Shanghai Normal University, Shanghai, China; ^4^CAS Center for Excellence in Molecular Plant Sciences, Shanghai Institute of Plant Physiology and Ecology, Chinese Academy of Sciences, Shanghai, China; ^5^Key Laboratory of Plant Resources Conservation and Sustainable Utilization, South China Botanical Garden, Chinese Academy of Sciences, Guangzhou, China; ^6^College of Science and Engineering, James Cook University, Cairns, QLD, Australia; ^7^Xiangxi Tujia and Miao Autonomous Prefecture Forest Resources Monitoring Center, Jishou, China; ^8^Research Center for Plants Conservation and Botanic Gardens, National Research and Innovation Agency of Indonesia, Bali, Indonesia

**Keywords:** Gleicheniaceae, cryptic diversity, species delimitation, phylogeny, taxonomy, new combination

## Abstract

Cryptic species comprise two or more taxa that are grounded under a single name because they are more-or-less indistinguishable morphologically. These species are potentially important for detailed assessments of biodiversity, but there now appear to be many more cryptic species than previously estimated. One taxonomic group likely to contain many cryptic species is *Dicranopteris*, a genus of forked ferns that occurs commonly along roadsides in Asia. The genus has a complex taxonomical history, and *D. linearis* has been particularly challenging with many intra-specific taxa dubiously erected to accommodate morphological variation that lacks clear discontinuities. To resolve species boundaries within *Dicranopteris*, we applied a molecular phylogenetic approach as complementary to morphology. Specifically, we used five chloroplast gene regions (*rbcL*, *atpB*, *rps4*, *matK*, and *trnL-trnF*) to generate a well-resolved phylogeny based on 37 samples representing 13 taxa of *Dicranopteris*, spanning the major distributional area in Asia. The results showed that *Dicranopteris* consists of ten highly supported clades, and *D. linearis* is polyphyletic, suggesting cryptic diversity within the species. Further through morphological comparison, we certainly erected ***Dicranopteris austrosinensis*** Y.H. Yan & Z.Y. Wei **sp. nov.** and ***Dicranopteris baliensis*** Y.H. Yan & Z.Y. Wei **sp. nov.** as distinct species and proposed five new combinations. We also inferred that the extant diversity of the genus *Dicranopteris* may result from relatively recent diversification in the Miocene based on divergence time dating. Overall, our study not only provided additional insights on the Gleicheniaceae tree of life, but also served as a case of integrating molecular and morphological approaches to elucidate cryptic diversity in taxonomically difficult groups.

## Introduction

Cryptic species, a common and increasingly used term, refers to taxa that are erroneously classified as a single species due to the paucity of conspicuous morphological differences (Trontelj and Fišer, 2009; Detwiler et al., 2010; Struck et al., 2018). Cryptic species represent a potentially important influence on the accuracy of detailed assessments of biodiversity (Witt et al., 2006; Trontelj and Fišer, 2009) and can lead to novel insights regarding patterns and processes of biodiversity, including geographic variation in species distributions and species coexistence (Fiser et al., 2018). However, cryptic species are seldom considered in biodiversity assessments owing to the lack of affordable and efficient diagnostic methods (Witt et al., 2006). This is compounded by the fact the high rate at which cryptic species are discovered in molecular studies suggests that number of cryptic species is far greater than prior estimates.

Studies on cryptic species throughout the whole tree of life have increased exponentially over the past two decades, fueled in large part by the increasing availability of DNA sequences, which facilitate various genetic approaches to the resolution of cryptic diversity (Sites and Marshall, 2003; Bickford et al., 2007). The prevalence of considerable cryptic diversity has been uncovered in a diverse range of groups, including in plants (Okuyama and Kato, 2009; Carstens and Satler, 2013; Ji et al., 2020; Kinosian et al., 2020; Li et al., 2020) and many animals (Hebert et al., 2004; Oliver et al., 2009; Manthey et al., 2011; Nadler and DE León, 2011; Phiri and Daniels, 2016), suggesting that cryptic species probably represent a significant portion of undiscovered biodiversity (Jörger and Schrödl, 2013; Pante et al., 2015; Loxdale et al., 2016). The ever-increasing cryptic diversity that genetics has resolved poses a taxonomic challenge in terms of what taxonomic ranks should be assigned to cryptic species that can be recognized on a genetic, but not necessarily morphological, basis. One practical proposal has been that the taxonomic ranks of species should be reserved for organisms showing observable morphological variation, while resolved cryptic species should be designated as intraspecific ranks (Maxwell and Dekkers, 2019; Maxwell et al., 2021).

**FIGURE 1:**
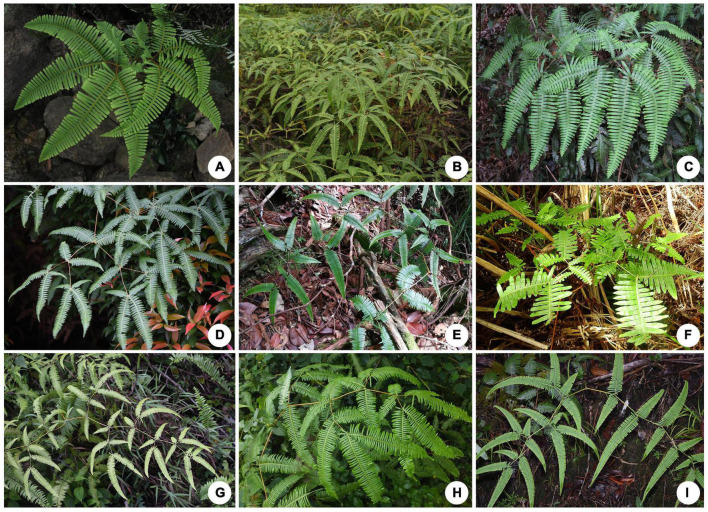
A selection of species of *Dicranopteris*. **(A)** *D. pedata*. **(B)** *D. linearis*. **(C)** *D. ampla*. **(D)** *D. tetraphylla*. **(E)** *D. inaequalis*. **(F)** *D. latiloba*. **(G)** *D. subpectinata*. **(H)** *D. alternans*. **(I)** *D. subspeciosa*.

Cryptic species may also be concealed in cases where there is considerable morphological variation but without clear boundaries supporting species delimitation, such as in the fern genus *Dicranopteris* Bernh. *Dicranopteris* is one of six genera in Gleicheniaceae (PPG I, 2016), an early-diverging leptosporangiate fern (Schuettpelz and Pryer, 2007; Choo and Escapa, 2018). The genus is unique on the part of its branching and leaf morphology as characterized by a pseudo-dichotomously branched fronds that produces a forking architecture, resulting from abortion or dormancy of the apical bud (Perrie et al., 2007). The unique morphology is attractive by which some species were used as ornamental plants (Marpaung and Susandarini, 2021). *Dicranopteris* is composed of about 20 species (Schuettpelz et al., 2016) that occur widely in tropical and subtropical areas (Ding et al., 2013). Across its range, *Dicranopteris* displays an extraordinarily high level of morphological diversity (Figure 1) that is not easily translated into species boundaries. In fact, due to these difficulties, no comprehensive species classification has been proposed for the genus, despite several prior studies based on morphology, sometimes with only regional sampling (Supplementary Table 1).

Within *Dicranopteris*, *D. linearis* (Burm.f.) Underw. has been particularly taxonomically challenging. *D. linearis* was divided into 13 varieties in Southeast Asia by Holttum (1959), who admitted that some varieties were more distinct than others and should probably be recognized as species. For example, the four varieties *D. linearis* var. *demota*, *D. linearis* var. *tetraphylla*, *D. linearis* var. *montana*, and *D. linearis* var. *altissima*, always possess accessory branches at the bases of their ultimate branches, whereas other varieties are not always present. In addition, these four varieties can be also separated based on other characteristics, such as branches at successive forks alternately being equal or unequal, lower pinnae surfaces bearing some hairs or not, angles of secondary and later forks, and dimensions of the pinnae (Underwood, 1907; Nakai, 1950; Ching et al., 1959; Holttum, 1959; Huang, 1994; Piggott, 1996; Ding et al., 2013). Following Holttum, Ding et al. (2013) recognized *D. linearis* and its varieties as a single species and placed them under the *D. pedata*, which likely obscured its real complexity. With these regards, taxonomic revisions on the placement of *D. linearis* and its varieties into the genus are very dynamic. Nonetheless, several varieties have been reinstated the status of species. A study by Ding et al. (2013) on the genus treated *D. linearis* var. *montana* under *D. taiwanensis*. Similarly, Kuo et al. (2019) in their phylogenetic study of updating Taiwanese pteridophyte checklist provided support for separating *Dicranopteris subpectinata* (Christ) C.M. Kuo and *Dicranopteris tetraphylla* (Rosenst.) C.M. Kuo of Taiwan from *D. linearis*.

Due to the diverse morphology and complex taxonomic history of *Dicranopteris* (Supplementary Table 1), we suspected that there may be a large number of cryptic species in this genus. Therefore, we sought to apply molecular phylogeny as a complement to morphology to resolve its species boundaries. To accomplish this, we incorporated 13 taxa, representing well over half of the known diversity in *Dicranopteris*, and five plastid DNA regions (*rbcL*, *atpB*, *matK*, *rps4*, and *trnL-trnF*). Specifically, our aims were to: (1) resolve phylogenetic relationships in *Dicranopteris*, especially among the varieties of *D. linearis*, (2) provide a preliminary revised taxonomy of *Dicranopteris* on the basis of molecular phylogeny and morphological characters, and (3) uncover cryptic diversity in the genus if present.

## Materials and Methods

### Taxon Sampling

We sampled 37 accessions representing 13 taxa of *Dicranopteris*, spanning the major centers of geographic distribution in Asia (Supplementary Table 2): China (23 accessions), Malaysia (7 accessions), Thailand (3 accessions), and Indonesia (4 accessions). Our sampling followed taxonomic treatments in Holttum (1959); Ding et al. (2013), and Schuettpelz et al. (2016). We also sampled close relatives for the outgroup: *Dipteris chinensis*, *Matonia pectinata*, *Sticherus truncatus*, and *Diplopterygium glaucum*. Notably, all samples were approved by for collection in China or provided by the collaborators in Malaysia, Indonesia, or Thailand. We deposited duplicates of all voucher specimens at the Shanghai Chenshan Herbarium (CSH) and the National Orchid Conservation Center of China and the Orchid Conservation and Research Center of Shenzhen (NOCC).

### DNA Extraction and Sequencing

We first extracted total genomic DNA from silica-gel dried leaves using a Plant Genomic DNA Kit (Tiangen Biotech, Beijing, China) following the manufacturer’s protocol. From the total DNA, we amplified five chloroplast DNA (cpDNA) regions including four coding regions (*rbcL, atpB, matK, rps4*) and one intergenic spacer (*trnL-trnF*). We performed polymerase chain reaction (PCR) amplification and sequencing for these gene regions using the primers shown in Table 1. The amplification procedure for all regions consisted of a 25-μl reaction volume containing 1–2 μl of template DNA, 1 μl each of 10 μM primers, 2.5 μl of 10× Taq Buffer with MgCl_2_, 0.2 μl of Taq polymerase, and ddH_2_O to volume. The amplification profiles comprised initial enzyme activation at 95°C for 5 min followed by 38 cycles of denaturation, primer binding, and extension at 94°C for 30 s, 58°C for 30 s, and 72°C for 1 min, respectively. The final extension was at 72°C for 10 min. The resulting PCR products were purified and sequenced in Sangon Biotech. All DNA sequences new to this study are available from GenBank (accession numbers in Supplementary Table 2).

**TABLE 1:** List of PCR amplification and sequencing primers used in the study.

Regions	Primer name	Primer sequence (5′–3′)	References
*rbcL*	F1	ATGTCACCACAAACGGAGAC	Li et al., 2004
	R1379	GCAGCTAATTCAGGACTCC	
*atpB*	F	AGCTTCATCGATGTTACC	Li et al., 2010
	R	GTTGGTGAAACTACTCTTGG	
*matK*	*mat*KrAGK	CGTRTTGTACTYYTRT GTTTRCVAGC	Kuo et al., 2011
	*matK*fEDR	ATTCATTCRATRTTTTTATTTH TGGARGAYAGATT	
*rps4*	F	ATGTCCCGTTATCGAGGACC	Li et al., 2010
	R	GGAATGATACTCGACGACTAG	
*trnL-trnF*	e	ATTTGAACTGGTGACACGAG	Small et al., 2005
	f	GGTTCAAGTCCCTCTATCCC	

### Phylogenetic Analyses

We edited and assembled the resulting sequences using SeqMan v7.1.0 (DNASTAR, United States) and aligned them using MUSCLE (Edgar, 2004) with default parameters followed by manual adjustment. Then, the alignments of the five cpDNA regions were concatenated into a single combined dataset using PhyloSuite (Zhang et al., 2020). Prior to performing phylogenetic analyses, we selected optimal partitioning strategies and best substitution models using PartitionFinder 2 (Lanfear et al., 2017) integrated into PhyloSuite, with three codon positions for each protein-coding gene provided as input partitions. The optimized partitioning schemes, and associated models are provided in Supplementary Table 1. We further utilized these optimized partitioning schemes and models to perform Bayesian inference (BI) and maximum likelihood (ML) analysis, respectively.

Maximum likelihood phylogeny was inferred using IQ-TREE (Nguyen et al., 2015) integrated into PhyloSuite under each edge-linked partition model with 5,000 ultrafast bootstrap replicates (Minh et al., 2013). For Bayesian inference, we performed the analyses in MrBayes v3.2.6 (Ronquist et al., 2012) with the optimized partitioning schemes and models. The BI analysis comprised two parallel runs of four Markov chain Monte Carlo (MCMC) each for 1,000,000 generations with every 1,000 generations sampled. The standard deviation of split frequencies was set to less than 0.01 to achieve the convergence of the independent runs. Following the analysis, we removed the first 25% of sampled generations as burn-in. Additionally, we carried out a maximum parsimony (MP) analysis on the concatenated dataset using MPBoot (Hoang et al., 2018) with 1,000 bootstrap replicates (*-bb 1,000*).

### Molecular Dating Analysis

The divergence time estimations were conducted using the concatenated cpDNA dataset in BEAST2 v2.6.3 (Bouckaert et al., 2019). We calibrated the BEAST analysis based on two fossils following Schuettpelz and Pryer (2009) suggesting ≥228 million years ago (Mya) as the divergence between Matoniaceae and Dipteridaceae along with ≥99.6 Mya for divergence of the clade consisting of *Sticherus* and *Diplopterygium*. We also used the results from Schuettpelz and Pryer (2009) to constrain the root of Gleicheniales (= 262.2 Mya) as a secondary calibration point with a normal distribution.

The Bayesian Evolutionary Analysis Utility (BEAUTi) (BEAST package) was utilized to generate an XML file for the analysis in BEAST, in which we applied a GTR model of nucleotide substitution with four gamma rate categories and an uncorrelated lognormally distributed relaxed (UCLD) model for the molecular clock. We ran the MCMC chain for 30 million generations with 25% burn-in and a sampling frequency of 30,000 generations. The tree branching process was inferred using the Yule model with other default settings. We used Tracer v1.7.1 (Rambaut et al., 2018) to check the effective sample size (ESS) of each parameter and found that all ESS exceeded 200, which is considered as recommended threshold to indicate stationarity (Drummond et al., 2012). We determined the maximum clade credibility tree using TreeAnnotator v2.6.3 (BEAST package) with median node heights and visualized it in FigTree v1.4.3 (Rambaut, 2016).

## Results

### Phylogenetic Relationships Within *Dicranopteris*

**FIGURE 2:**
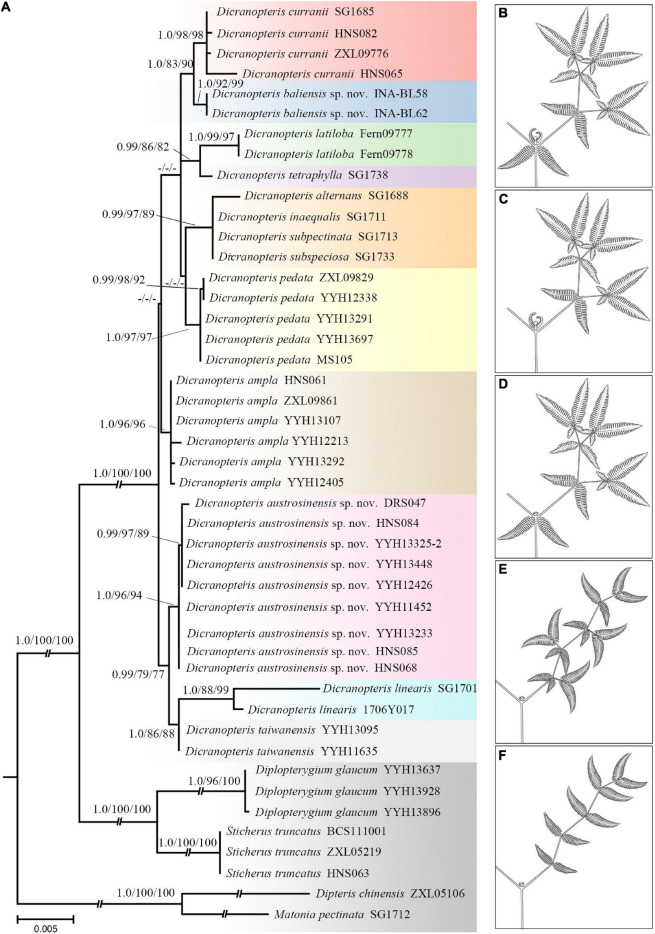
It showing phylogeny and the main morphological diagnostic features of *Dicranopteris* (drawn by LJC). **(A)** ML tree of *Dicranopteris* based on the concatenation of *rbcL, atpB, rps4, matK*, and *trnL-trnF*. Numbers above each branch are support values in the order of posterior probability from Bayesian analysis (BIPP), bootstrap percentages of maximum likelihood analysis (MLBS), and bootstrap percentages of maximum parsimony (MPBS). The dash (-) indicates a node with BIPP < 0.9, MLBS < 70%, MPBS < 70%. **(B)** A latend bud with stipule-like pseudostipules always presents at the primary fork and ultimate fork. **(C)** The primary fork with terminal dormant buds and stipule-like pseudostipules. **(D)** The primary fork with a sterile bud. **(E)** Lateral branches forked more than three times and alternate unequal forking. **(F)** Lateral branches forked more than three times and without accessory branch.

In order to reveal the phylogenetic relationships between nine species of *Dicranopteris* along with its four varieties, a molecular phylogenetic analysis was performed based on five DNA regions. The concatenated cpDNA sequences yielded an aligned data matrix 3,430 bp long. Phylogenetic analyses using BI, ML, and MP showed that *Dicranopteris* comprises 10 highly supported clades (Figure 2 and Supplementary Figures 1, 2). Accessions of widely accepted species of *Dicranopteris*, *D. ampla*, and *D. pedata*, formed well-resolved clades (BIPP = 0.99, MLBS = 96%, and MPBS = 96%; BIPP = 0.99, MLBS = 98%, and MPBS = 96%; respectively). Moreover, *D. gigantea* and *D. curranii*, collected from Hainan Island of China (HNS065 and HNS082) as well as Thailand (ZXL09776) and Malaysia (SG1685) formed a clade (BIPP = 1.0, MLBS = 98%, and MPBS = 98%) (Figure 2 and Supplementary Figures 1, 2). The analyses revealed that *D. linearis* was polyphyletic with several varieties clustered with other species of forked ferns (Figure 2 and Supplementary Figures 1, 2). Notably, accessions of *D. linearis* from southern China formed a clade that was strongly supported as a sister to *D. linearis* from Malaysia and *D. taiwanensis* (BIPP = 0.99, MLBS = 79%, and MPBS = 77%). Additionally, a species similar to that of *D. curranii* formed a highly supported (BIPP = 1.0, MLBS = 92%, and MPBS = 99%), which has a range within the Indochina Peninsula (Figure 2 and Supplementary Figures 1, 2).

### Divergence Time Estimation

**FIGURE 3:**
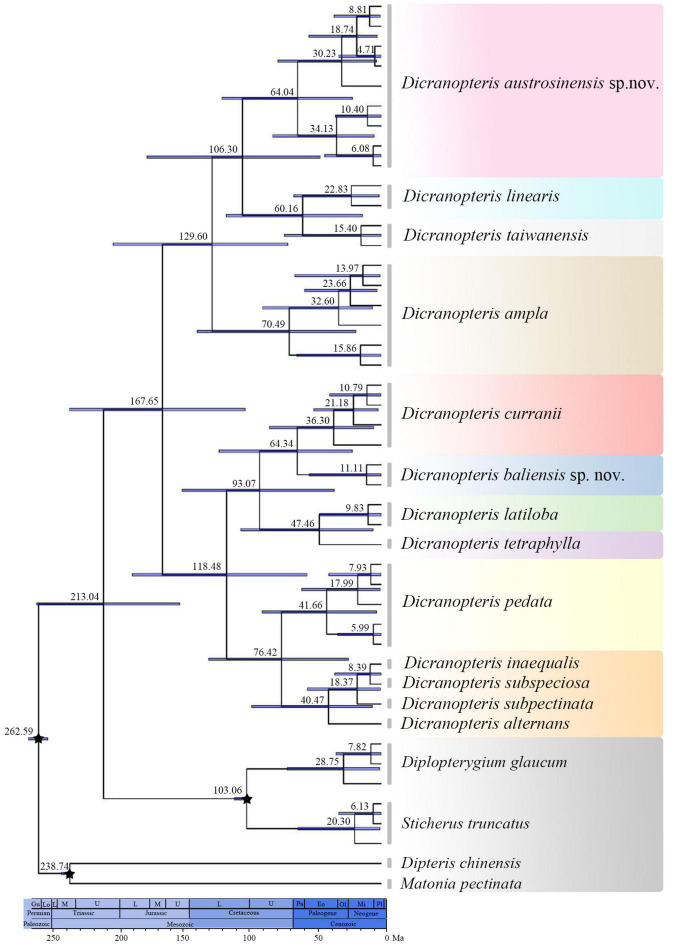
Chronogram presenting estimated divergence times using BEAST based on the concatenated *rbcL*, *atpB*, *rps4*, *trnL-trnF*, and *matK* dataset. Numbers above/under the tree branches represented median ages and 95% highest posterior density (HPD) intervals. Break stars indicated the calibrating points.

Our divergence time estimations indicated that *Dicranopteris* evolved ca. 213.04 Mya [95% highest posterior density (HPD): 154.65–264.09 Mya] (Figure 3). The stem age of *Dicranopteris* was estimated to be approximately 167.65 Mya (95% HPD: 104.25–238.95 Mya) (Figure 3). *Dicranopteris* diversified considerably from the Paleocene to Miocene (84.33–9.99 Mya) (Figure 3), and many varieties of *D. linearis* originated during that time.

## Discussion

### Hidden Cryptic Biodiversity in *Dicranopteris*

Evolutionary stasis is common for ancient lineages (Bomfleur et al., 2014; Jin et al., 2020; Wei et al., 2021), such as ferns. Accordingly, cryptic species may be particularly prevalent among these lineages due to extensive phenotypic plasticity, often resulting in extreme difficulty in classifying old taxa (Li et al., 2020). In this study, we found that the main extant lineages of *Dicranopteris* were ancient (Figure 3), which is consistent with the cryptic diversity in the genus that we detected as well as ongoing taxonomic difficulties. Compounding the problem is that *Dicranopteris* may have experienced flourishing diversification during the Paleocene to the Micoene (Schneider et al., 2004; Schuettpelz and Pryer, 2007; Sundue et al., 2015; Figures 2, 3 and Supplementary Figure 1), and this can also lead to subsequent taxonomic confusion.

**FIGURE 4:**
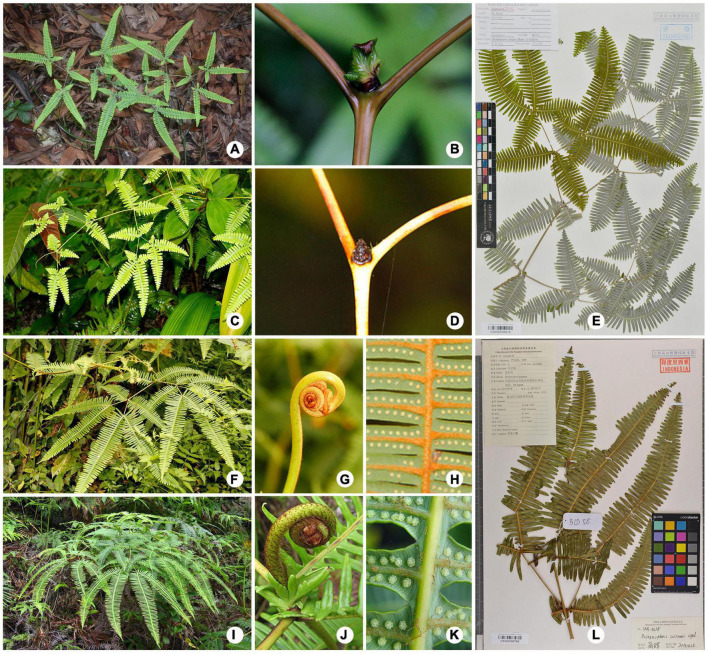
It showing two new species and similar species. **(A)** *D. austrosinensis* sp. nov. **(B)** The main fork of *D. austrosinensis* sp. nov. showing a latend bud with stipule-like pseudostipules. **(C)** *D. taiwanensis*. **(D)** The main fork of *D. taiwanensis* showing sterile bud. **(E)** The type specimen of *D. austrosinensis* sp. nov. **(F)** *D. baliensis* sp. nov. **(G)** Crozier of *D. baliensis* sp. nov. with red–brown scales. **(H)** Lower surface of costae and costules of *D. baliensis* sp. nov. with rather persistent hairs. **(I)** *D. curranii*. **(J)** Crozier of *D. curranii* with atrocastaneous scales. **(K)** Lower surface of costae and costules of *D. curranii* with deciduous hairs. **(L)** The type specimen of *D. baliensis* sp. nov.

Recently, Marpaung and Susandarini (2021) investigated the variation on morphology and spore characters among two species and seven varieties of *Dicranopteris*. The result revealed that the genus has high morphological variations, as indicated by the presence of intraspecific categories. In the present study, we further used molecular data as complementary to morphology to explore cryptic biodiversity in *Dicranopteris*. We were able to phylogenetically and morphologically differentiate two new species (Figures 2, 4), *D. austrosinensis* sp. nov. and *D. baliensis* sp. nov., and resolve several previously unsettled aspects of species classification of *Dicranopteris*, including five new combinations. In addition, the species *D. pedata* recorded in Flora of China (Ding et al., 2013) was proved to hide cryptic species. Specifically, the current treatment of *D. pedata* in Flora of China consists of three species, *D. linearis*, *D. austrosinensis* sp. nov., and *D. pedata* s. str. Among the three species, only *D. austrosinensis* sp. nov. presents a pair of accessory branches at the ultimate branches. *D. linearis* differs from the similar *D. pedata* by having more basal inner segments of ultimate pinnules shortened. Overall, we have preliminarily revealed that *Dicranopteris* likely harbors a large constellation of cryptic diversity (Figure 2 and Supplementary Figures 1, 2).

### Preliminary Phylogenetic Analyses

The molecular phylogenetic relationships of *Dicranopteris* have never been comprehensively investigated in prior studies. Previous studies have included only several well-accepted species of the genus, such as *D. pedata* and *D. linearis* (Hasebe et al., 1995; Schuettpelz and Pryer, 2007), within studies of broader taxonomic groups. In this study, we included accessions representing well over half of the currently recognized diversity of *Dicranopteris* and utilized three methods of phylogenetic reconstruction (ML, BI, and MP).

The preliminary phylogenetic analyses showed a clear division of species in *Dicranopteris*, which was generally congruent with previous study based on morphology (Ching et al., 1959), except for varieties of *D. linearis*. *D. linearis* exhibited no close relationship with its varieties, while was typically well supported as monophyletic with *D. taiwanensis* (BIPP = 1.0, MLBS = 86%, and MPBS = 88%) (Figure 2 and Supplementary Figures 1, 2). In addition, *D. linearis* was proved to be a distinct species and not a synonym for *D. pedata* as previously thought (Ding et al., 2013; Figure 2 and Supplementary Figures 1, 2). For the four species (i.e., *D. alternans*, *D. inaequalis*, *D. subspeciosa*, and *D. subpectinata*), the species status and phylogenetic relationship were not well-documented and untangled up in the present study (Figure 2 and Supplementary Figures 1, 2) due to in the absence of adequate samples. Conversely, they can be identified by many morphological characters, such as angle of the forks, lower surface covering, etc. Nonetheless, we believe that classifying them would be premature, as we seek stable taxonomic solutions within the genus and for the *D. linearis* complex.

In a broad sense, the phylogenetic positions of several main clades of *Dicranopteris* had low support (i.e., BIPP < 0.90, MLBS < 70%, and MPBS < 70%). Consequently, in the future, more molecular markers and denser sampling of taxa should be included to better resolve taxonomic relationships. Also, a more comprehensive systematic investigation of *Dicranopteris* should accompany the ongoing work on the Flora of Asia to detect and account for cryptic diversity in the genus.

## Conclusion

The present study represented the first comprehensive molecular phylogenetic analysis of *Dicranopteris* and, therefore, provided additional insights on the Gleicheniaceae tree of life. On the basis of our molecular phylogeny from cpDNA and morphological evidence, we showed that *Dicranopteris* hides considerable cryptic diversity, and we were able to disentangle two new cryptic species, *D. austrosinensis* sp. nov. and *D. baliensis* sp. nov., from the diverse species complex, *D. linearis*. We also clarified the previously unsettled status of five taxa in *Dicranopteris* and, thus, proposed new combinations. We found that *Dicranopteris* evolved 213.04 Mya and underwent a flourishing diversification during the Miocene leading to much of its extant diversity. Our study highlighted the importance of applying morphology and molecular data to resolve relationships within taxonomically recalcitrant genera and seek out cryptic diversity. Within *Dicranopteris*, our results probably represented only the “tip of the iceberg” in terms of cryptic diversity, and much more intensive sampling is critical in future studies.

## Taxonomic Treatment

(1) ***Dicranopteris austrosinensis*** Y.H.Yan & Z.Y.Wei, sp. nov.

Type: —CHINA, Hong Kong, Mt. Parker, October 26, 2010, *Hong Kong Fern Survey Team E016* (holotype, CSH).

Diagnosis: —The new species is similar to *Dicranopteris taiwanensis* in having a pair of accessory branches at ultimate branches, while it differs in the main forks presenting a latent bud with two lobed pseudostipules.

Description: —**Plants** terrestrial, about 1.5 m tall. **Rhizome** creeping, (2–)3–5 mm thick, covered with dense ferrugineous hairs. **Rachis** dark brown, glabrous, 0.7–1 m long, 2–6 mm thick, 4–5 times dichotomously branched, forking in lateral branch–systems alternately unequal, fractiflex, with a pair of lateral stipule-like pinnules at each dichotomy except for the primary branch, basal internode 7–10 cm, second internode slightly shorter, 6–7 cm long, ultimate internode shortest, usually 4–5 cm long. **Dormant buds** ovate, ca. 4–8 × 5–10 mm, covered with brown hairs, subtending pseudostipules small, lanceolate with rounded bases, margin irregularly triangular. **Pinnae** 4–5 forked, lanceolate or broadly lanceolate, a pair of lateral pinnae at each dichotomy lanceolate shorter, 8–12 cm long and 4–6 cm wide, rest pair of pinnae large, 3–4 × 13–16 cm, base and apex attenuate, abaxially glabrous and pale, yellowish green or green adaxially, 2–3 pairs of basal segments decrescent. **Segments** 2–3 cm, 20–35 pairs, oblong to cylindrical, apex truncate, adaxially glabrous, the veins 2–3 forked. **Sori** inframedial, 1 line on each side of costule, 8–10 sporangia per sorus.

Additional Specimens Examined: —**China.** Guangdong Province. Shenzhen City, Dapeng New District, Yangmeikeng, 16 August 2020, *Yan Y.H. et al. YYH15634* (NOCC); Gaoyao City, 1 August 1932, *Lau S.Y. 20333* (PE); Zhaoqing City, Deqing District, 16 July 1958, *Liu H.G. 00904* (PE). Hainan Province. Changjiang Li Autonomous County, Bawangling, 4 April 2002, *Chen Z.C. 010796* (SZG). Hong Kong. 27 September 2002, *Yan Y.H. 665* (IBSC). Guangxi Province. Guangxi Zhuang Autonomous Region, Fangkonggang City, Fangcheng District, Nawan mountain, 14 August 2010, *Xie Y.J.* & *Liang S.Z. B0822* (IBK). Guizhou Province. Qiannan Buyi and Miao Autonomous Prefecture, Libo District, 27 April 2015, *Zhang X.C., Guo Z.Y.* & *Xiang Q.P. 7297* (PE). **Vietnam**. Mount Tai Hoang Mao, 30 July 1939, *Tseng H.T. 29363* (HITBC, photo!).

Distribution and ecology: —It forms dense colonies in open habitats in montane forests, thickets and forest margins, at ca. 50–500 m. Mainly occurring in South China (Guangxi, Guangdong, Hainan, Hong Kong, Guizhou) and Vietnam.

(2) ***Dicranopteris baliensis*** Y.H.Yan & Z.Y.Wei sp. nov.

Type: —Indonesia, Bali Island, September 29, 2014, *Yan Y.H.* & *Shang H. INA-BL58* (holotype, CSH).

Diagnosis: —The new species is similar to *Dicranopteris curranii* with large fronds, rachis 2 or 3 times dichotomously branched, and sori in 1 line on each side of costule, but differs in lower surface of costae and costules densely hairy, red–brown, and persistently.

Description: —**Plants** terrestrial, 1–1.5 m tall. **Rhizome** creeping. **Rachis** stamineous, moderately brown scales, ca. 30–50 cm long between pinnae, 2 or 3 times dichotomously branched, primary rachis-branches almost equally forked, first branch 2.5–3.8 cm long and 1.0–1.8 mm wide, second branch shorter, 1.8–2.2 cm long and 0.8–1.0 mm wide, opposite branches of equal length, accessory branches at each dichotomy except for the ultimate branch, distal branches with overlapping segments. **Rachis buds** covered with short and usually concolorous red–brown scales. **Dormant buds** ovate, ca. 3–5 × 8–10 mm, covered with dense red–brown hairs, pseudostipules lanceolate. **Pinnae** lanceolate, generally large, ultimate pinnae longest, 25–30 cm long, 8–10 cm wide, basal pairs of segments slightly narrowed, apex acuminate, 7–8 pairs of distal segments decussate, with red–brown hairs abaxially. **Segments** 50–52 pairs, 2.2–3 cm long, 4.1–6.8 mm wide, distal and medial pairs of segments largest, the veins 2–3 forked. **Sori** inframedial, 1 line on each side of costule, 12–14 sporangia per sorus.

Additional Specimens Examined: —**Indonesia.** Bali Island, September 29, 2014, *Yan Y.H.* & *Shang H. INA-BL62* (CSH).

Distribution and ecology: —Terrestrial in thickets, forest, forest margins. Endemic to montane forests in Indonesia (Bali Island).

### Further Taxonomic Novelties

(1) ***Dicranopteris inaequalis*** (Rosenst.) Y.H.Yan & Z.Y.Wei, comb. & stat. nov.

≡ *Gleichenia linearis* var. *inaequalis* Rosenst., Fedde, Rep. 13 (1915) 212.

≡ *Dicranopteris linearis* var. *inaequalis* (Rosenst.) Holtt., Reinwardtia 4 (1957) 278.

Type: —Indonesia. Sumatra, Batakerland, January 1, 1911, *Winkler, K.J.M. 114* (S, S-P-4212, photo!).

Notes: —*Dicranopteris inaequalis* is closely related and similar to *D. subspeciosa*, but is recognized by having very unequal lateral branch-systems. *D. inaequalis* often dichotomously forks 4 or 5 times and angle of the secondary and later rachis forking more than a right angle. The smaller branches at once forked occasionally lack an accessory branch. Pinnae are lanceolate or broadly lanceolate and have 2–4 pairs of basal decrescent segments. Segments are 1.5–2 mm wide, 1.5–2 cm long, and covered with pale hairs.

Distribution and ecology: —*Dicranopteris inaequalis* is mainly distributed in Malay Peninsula. It is locally common along roadsides, slopes and forest edges at ca. 1200–1400 m.

Specimen examined: —**Malaysia.** Johor Mount Ophir, May 20, 2017, *Yan Y.H.* & *Shang H. SG1711* (CSH). **Indonesia.** Sumatra, Batakerlande, January 1, 1911, *Winkler, K.J.M. 114* (UC, UC391814, photo!). **Papua New Guinea**. September 26, 1964, *TREVOR G. WALKER, T. F820* (UC, photo!).

(2) *Dicranopteris alternans* (Mett.) Y.H.Yan & Z.Y.Wei, comb. & stat. nov.

≡ *Gleichenia dichotoma* var. *alternans* Mett., Miq. Ann. Mus. Bot. Lugd. Bat. 1 (1863) 51.

≡ *Gleichenia linearis* var. *alternans* Alderw., Malayan Ferns Fern Allies. (1917) 84.

≡ *Dicranopteris linearis* var. *alternans* (Mett.) Holtt., Reinwardtia 4 (1957) 278.

Lectotype: —Indonesia. Sumatra, Korthals (L, L0051519, photo!).

Isolectotypes: —Indonesia. Sumatra, Korthals (S, S-P-4210, photo!); Sumatra, Korthals (S, S-P-4209, photo!).

Notes: —*Dicranopteris alternans* is a relatively little common species. It is similar to *D. subpectinata*, and the most important difference between them is the indument on the lower surface of lamina. In *D. alternans* the lower surface of lamina bears copious red–brown hairs, whereas quite glabrous in *D. subpectinata*. *D. alternans* has rather firm lamina as well as broad–lanceolate and large pinnae with attenuate apex. Ultimate pinnae are 20–30 cm long and 4–5 mm wide. Lateral branches usually fork more than three times, and the branches at successive forks are alternately unequal.

Distribution and ecology: —Rare in open habitats in the lowlands and on the edge of forest in the hills at 300–500 m in Malaysia, Indonesia, Sumatera, Singapore, Borneo, and Banka.

Specimen examined: —**Malaysia.** January 28, 1959, *Hassan, N.* & *Kadim, H. 102* (L, photo!); **Indonesia.** January 26, 1958, *Holttum, R.E. s.n.* (L, photo!); February 11, 1920, *Bünnemeijer, HAB 8165* (L, photo!); October 22, 1917, *Bünnemeijer, HAB 1657* (L, photo!); *Korthals, P.W. s.n.* (K, photo!); *Korthals, P.W. 113* (L); April 13, 1923, *Lörzing, JA 9620* (L, photo!); Sumatra, Harau-canyon near Pajakumbuh, January 25, 1958, *W. Meijer 7527* (K, photo!); Sumatra, Korthals, P.W., # [115] s.n. **Singapore.** November 12, 1956, *Bels, L 271* (B, photo!). **Samoa.** October 1, 1893, *Reinecke, F 82* (L, photo!). **UK.** Royal Botanic Garden Kew, s. coll. K000377055 (K).

(3) *Dicranopteris subspeciosa* (Holtt.) Y.H.Yan & Z.Y.Wei, comb. & stat. nov.

≡ *Dicranopteris linearis* var. *subspeciosa* Holtt., Reinwardtia 4 (1957) 278.

Type: —**Malaysia.** Borneo, Sabah, Kiau, Mt Kinabalu, October 31, 1915, *Topping, D.L. 1516* (US, US00134664, photo!).

Notes: —*Dicranopteris subspeciosa* is also an uncommon species. It is characterized by having alternately unequal branches with the angle of the ultimate fork being 90° or less than 90°. The basal two branches are of roughly similar length that may be up to twice of the same length as the ultimate branches. The species has the smallest pinnae, 13–15 cm long, 4–5 cm wide. The pinnae are long–lanceolate with caudate apex. Typically, *D. subspeciosa* has pale, slender and entangled hairs covering the lower surface of lamina.

Distribution and ecology: —Best known in Malaysia, where it grows along roadsides and on the edge of forest, but also known from scattered specimens from Philippines.

Specimen examined: —**Malaysia.** Borneo, Sabah, Kiau, Mt Kinabalu, October 31, 1915, *Topping, D.L. 1516* (isotype, MICH, photo!); October 28, 1915, *Clemens, MS 9789* (MICH, photo!); May 1910, *Brooks, CJ 152* (MICH, photo!); Borneo, Sabah, Kiau, Mt Kinabalu, November 2, 1980, *Parris, B.S.* & *Croxall, J.P. 8980* (K); Kota Kinabalu, Signal Hill, September 17, 1975, *Shim, P.S. s.n.* (K). **Philippines.** June 10, 1953, *Edaño, GE 6366* (MICH, photo!); October 1912, *Elmer, ADE 14142* (MICH, photo!); March 22, 1953, *Edaño, GE 6104* (MICH, photo!). May 21, 1946, *Alcasid, GL 286* (MICH, photo!); July 1917, *Elmer, ADE 7875* (MICH, photo!).

(4) *Dicranopteris latiloba* (Holtt.) Y.H.Yan & Z.Y.Wei, comb. & stat. nov.

≡ *Dicranopteris linearis* var. *latiloba* Holtt., Reinwardtia 4 (1957) 277.

Type: —Philippines. Luzon, Benguet, May 1911, *Merrill, E.D. 975* (US, US00134663, photo!).

Notes: —*Dicranopteris latiloba* is one of the species with the widest segments (ca. 5–6 mm) in the genus. It is charactered by lacking a pair of accessory branches at ultimate forks, by having deeply and broadly lobed segments and quite glabrous lower surface of lamina. Usually, *D. latiloba* is dwarf, lateral branches forked about 1–3 times. Branches at all forks are subequal with somewhat irregularly placed segments. Segments are oblong or emarginate, firm but not very thick, reflexed when dry. Typically, the angle of ultimate fork with no accessory branches is approximately a right angle.

Distribution and ecology: —It is locally common in Malaysia and Philippines. Growing in montane forests at 1000–1500 m.

Specimen examined: —**Philippines.** Luzon, Benguet, May 1911, *Merrill, E.D. 975* (isotype, MICH, MICH1190343, photo!); May 16, 1948, *Edaño, GE PNH 5231* (L, photo!); June 1, 1917, *Elmer, ADE 17873* (L, photo!); May 1911, *Phil. Plts. 975* (L, photo!); June 1, 1908, *Elmer, ADE 10351* (L, photo!); May 1, 1907, *Elmer, ADE 9031* (L, photo!). **India.** November 29, 1945, *Hou H.Y. 5113* (PE, photo!).

(5) ***Dicranopteris curranii*** Copel, Philip. J. Sc. 81 (1952) 4.

**Type:** —Philippines. Luzon, Laguna, February 1910, *Curran, H.M. BS19265* (K, K000399480, photo!).

= *Dicranopteris gigantea* Ching Fl. Reipubl. Popularis Sin. 2 (1959) 346. ***syn. nov.***

Notes: —This name was coined by Copel based on a collection from Curran. Our study has convinced us that *D. curranii* is conspecific with *D. gigantea* named by Ching et al. (1959). Because *D. curranii* was published before *D. gigantea*, we therefore consider that *D. gigantea* should be treated as a synonym of *D. curranii*. *D. curranii* is larger as compared to any other species in the genus. Rachis dichotomously fork 2 or 3 times, with a pair of lateral pinnules at each dichotomy. It has the longest and widest pinnae. Ultimate pinnae are commonly 40–60 cm long and 13–15 cm wide, lanceolate, long caudate apex. Segments are 5–8 cm long, 3.5–4.5 mm wide, linear, reflexed when dry. Lower surface of costae and costules present sparse, brown, and deciduous hairs. Fiddleheads are covered with dense brown scales.

Distribution and ecology: —Southern China, Malaysia, Philippines, Indonesia, Thailand, and Vietnam. It grows in thickets, along roadsides and in other open habitats in montane forests at below 1500 m.

Specimen examined: —**Philippines.** February 1910, *Curran, H.M. BS19265* (US, photo!). **Malaysia.** August 17, 1983, *Zogg, E. 7045* (L, photo!); October 14, 1967, *Shimizu, T* & *Iwatsuki, K* & *Fukuoka, N* & *Hutoh, MM 13048* (L, photo!); September 2, 1971, *Iwatsuki, K* & *Murata, G* & *Dransfield, J* & *Saerudin, DS 1433* (L, photo!). **Indonesia.** August 17, 1973, *Murata, G* & *Fukuoka, N* & *Sukasdi J- 620* (L, photo!); September 5, 1919, *Bünnemeijer, HAB 7769* (L, photo!); March 24, 1894, *Schiffner, VF P 87* (L, photo!). **China.** Yunnan Province. Hekou Yao Autonomous County, October 30, 1989, *Zhu W.M. 23468-24* (HGAS), *Zhu W.M. 23468- c-1* (HGAS); Hekou Yao Autonomous County, September 11, 1962, *Wu, S.G. 4082* (PE). Hainan Province. Lingshui Li Autonomous County, December 20, 2006, *Yan Y.H. 3489* (HUST); Yingge Ling National Nature Reserve, March 28, 2015, *Shang, H., Wei, H.J., Shu, J.P.* & *Huang, K.R. SG2761* (CSH); July 19, 2018, *Wu, L. 6667* (CSFI). **Thailand.** Phangnga Province, July 21, 1999, *Watthana, S. et al., 457* (QBG); Pheaburi Province, December 19, 2014, *Zhou, X.L., Yan, Y.H.* & *Du, C. ZXL09776* (CSH). **Vietnam.** June 1, 2002, *Wu, S.G., Peng, H. et al., T-V467* (KUN); Lào Cai Province, Van Bàn County, June 1, 2006, *Wu, S.G., Peng, H. et al., T-V467* (KUN, KUN 1212243, photo!); Lào Cai Province, Van Bàn County, June 1, 2006, *Wu, S.G., Peng, H. et al., T-V467* (KUN, KUN 1212242, photo!).

## Key to *Dicranopteris* Species of Asia

1a. Ultimate forks with a pair of accessory branches …………..2
1b. Ultimate forks without a pair of accessory branches………4
2a. Main fork presenting a sterile bud without pseudostipules………………………………………….. *D. taiwanensis*
2b. Main fork showing a latent bud with two pseudostipules…………………………………………………………………3
3a. Leaves leathery, latent bud with two pinnatifid pseudostipules at mainforks ………………………. *D. tetraphylla*
3b. Leaves papery, latent bud with two lobed pseudostipules at main forks …………………………………………….. *D. austrosinensis*
4a. Lateral branches at each fork approximately equal; ultimate pinnae 10-15 cm wide …………………………………………………… 5
4b. Lateral branches at each fork alternately unequal; ultimate pinnule 3-6 cm wide ……………………………………………………… 7
5a. Lower surface of costae and costules glabrous……….. *D. ampla*
5b. Lower surface of costae and costules hairy……………………..6
6a. Lower surface of costae and costules sparsely hairy, brown, deciduously…………………………………………………….*D. curranii*
6b. Lower surface of costae and costules densely hairy, red-brown, persistently…………………………………………*D. baliensis*
7a. Lateral branches forked 1-3 times…………………………………8
7b. Lateral branches forked more than 3 times………………….10
8a. Angle of ultimate fork approximately a right angle………………………………………………………………*D. latiloba*
8b. Angle of ultimate fork usually less than 60°……………………9
9a. Ultimate branches with only 3-4 basal internal segments gradually reduced………………………………………….*D. pedata*
9b. Ultimate branches with approximately 10 basal internal segments gradually reduced………………………………*D. linearis*
10a. Lower surface glabrous…………………………….*D. subpectinata*
10b. Lower surface covered with hairs…………………………………11
11a. Lower surface of lamina segments with red-brown hairs…………………………………………………………….*D. alternans*
11b. Lower surface of lamina segments with pale hairs………….12
12a. Angle of the secondary and later rachis forking not more than a right angle…………………………………………*D. subspeciosa*
12b. Angle of the secondary and later rachis forking more than a right angle…………………………………………………..*D. inaequalis*

## Data Availability Statement

The datasets presented in this study can be found in online repositories. The names of the repository/repositories and accession number(s) can be found in the article/Supplementary Material.

## Author Contributions

ZYW and YHY designed the experiments. ZYW, ZQX, and JPS performed the research. HS, XLZ, WX, BA, and YHY collected the materials. ZYW and ZQX analyzed the data. ZYW and LJC drew the figures and wrote the draft. QY processed the figures. YHY, SM, and JGC revised the draft. All authors contributed to the article and approved the submitted version.

## Conflict of Interest

The authors declare that the research was conducted in the absence of any commercial or financial relationships that could be construed as a potential conflict of interest.

## Publisher’s Note

All claims expressed in this article are solely those of the authors and do not necessarily represent those of their affiliated organizations, or those of the publisher, the editors and the reviewers. Any product that may be evaluated in this article, or claim that may be made by its manufacturer, is not guaranteed or endorsed by the publisher.
