# Corrigendum: Phylogeny and Taxonomy on Cryptic Species of Forked Ferns of Asia

**DOI:** 10.3389/fpls.2022.888725

**Published:** 2022-04-12

**Authors:** Zuoying Wei, Zengqiang Xia, Jiangping Shu, Hui Shang, Stephen J. Maxwell, Lijun Chen, Xile Zhou, Wang Xi, Bayu Adjie, Quan Yuan, Jianguo Cao, Yuehong Yan

**Affiliations:** ^1^Key Laboratory of National Forestry and Grassland Administration for Orchid Conservation and Utilization, The National Orchid Conservation Center of China and The Orchid Conservation and Research Center of Shenzhen, Shenzhen, China; ^2^Eastern China Conservation Centre for Wild Endangered Plant Resources, Shanghai Chenshan Botanical Garden, Shanghai, China; ^3^College of Life Sciences, Shanghai Normal University, Shanghai, China; ^4^CAS Center for Excellence in Molecular Plant Sciences, Shanghai Institute of Plant Physiology and Ecology, Chinese Academy of Sciences, Shanghai, China; ^5^Key Laboratory of Plant Resources Conservation and Sustainable Utilization, South China Botanical Garden, Chinese Academy of Sciences, Guangzhou, China; ^6^College of Science and Engineering, James Cook University, Cairns, QLD, Australia; ^7^Xiangxi Tujia and Miao Autonomous Prefecture Forest Resources Monitoring Center, Jishou, China; ^8^Research Center for Plants Conservation and Botanic Gardens, National Research and Innovation Agency of Indonesia, Bali, Indonesia

**Keywords:** Gleicheniaceae, cryptic diversity, species delimitation, phylogeny, taxonomy, new combination

In the original article, there were three errors. The authors of the basionym for the “*Dicranopteris alternans* (Rosenst.) Y.H.Yan & Z.Y.Wei, comb. & stat. nov.,” “*Dicranopteris subspeciosa* (Rosenst.) Y.H.Yan & Z.Y.Wei, comb. & stat. nov.,” and “*Dicranopteris latiloba* (Rosenst.) Y.H.Yan & Z.Y.Wei, comb. & stat. nov.” were incorrect in the section **Further Taxonomic Novelties**. The authors of the basionym were corrected to “*Dicranopteris alternans* (Mett.) Y.H.Yan & Z.Y.Wei, comb. & stat. nov.,” “*Dicranopteris subspeciosa* (Holtt.) Y.H.Yan & Z.Y.Wei, comb. & stat. nov.,” and “*Dicranopteris latiloba* (Holtt.) Y.H.Yan & Z.Y.Wei, comb. & stat. nov.,” respectively.

The correct headings for the section **Further Taxonomic Novelties** appear below:

(2) *Dicranopteris alternans* (Mett.) Y.H.Yan & Z.Y.Wei, comb. & stat. nov.

(3) *Dicranopteris subspeciosa* (Holtt.) Y.H.Yan & Z.Y.Wei, comb. & stat. nov.

(4) *Dicranopteris latiloba* (Holtt.) Y.H.Yan & Z.Y.Wei, comb. & stat. nov.

The authors apologize for this error and state that this does not change the scientific conclusions of the article in any way. The original article has been updated.

## Publisher's Note

All claims expressed in this article are solely those of the authors and do not necessarily represent those of their affiliated organizations, or those of the publisher, the editors and the reviewers. Any product that may be evaluated in this article, or claim that may be made by its manufacturer, is not guaranteed or endorsed by the publisher.

