# Supplementary material for: Phylogeny and Taxonomy on Cryptic Species of Forked Ferns of Asia
Source: Front Plant Sci. 2021 Dec 17;12:748562. doi: 10.3389/fpls.2021.748562 (PMC8718997; doi:10.3389/fpls.2021.748562)
Supplement: Supplementary file 1 [file Data_Sheet_1.docx]

Supplementary Material

# Supplementary Figures and Tables

## Supplementary Figures


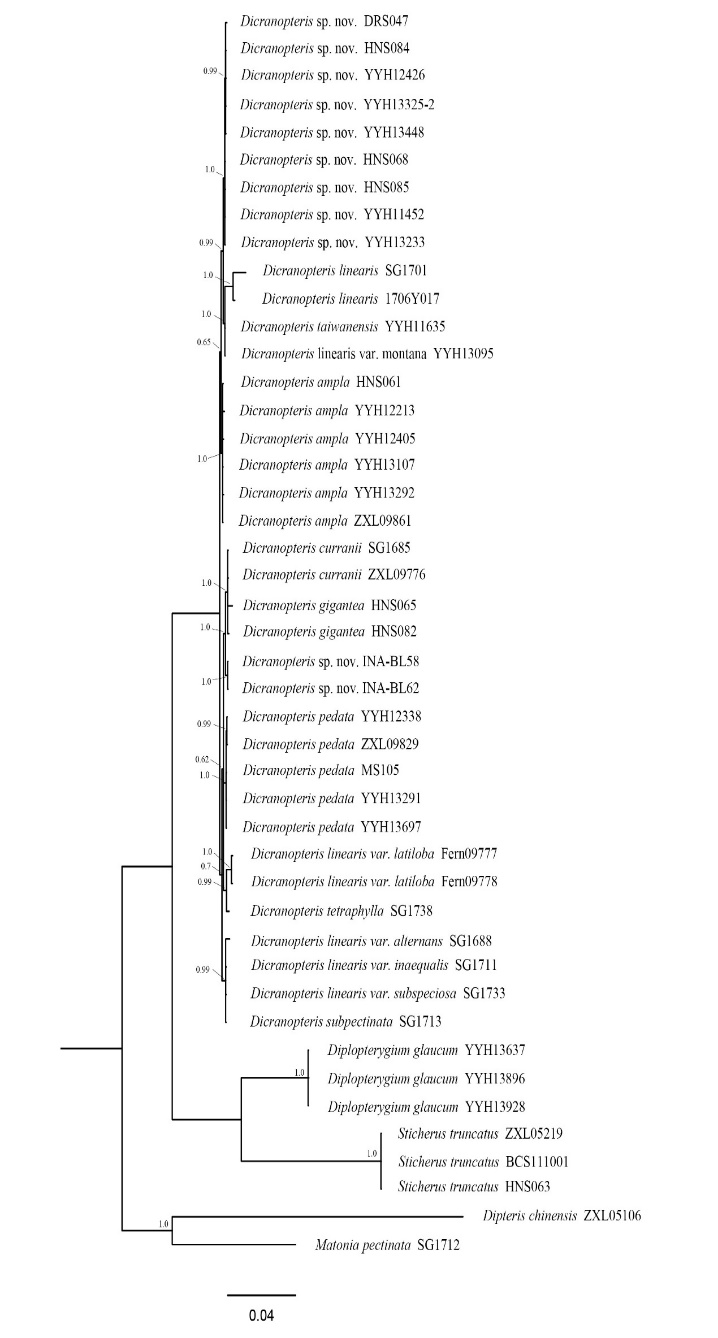


**Supplementary Figure 1.** The phylogenetic topology of *Dicranopteris* based on the concatenated dataset including sequences of *rbcL*, *atpB*, *rps4*, *trnL-trnF* and *matK* from Mrbayes analysis.


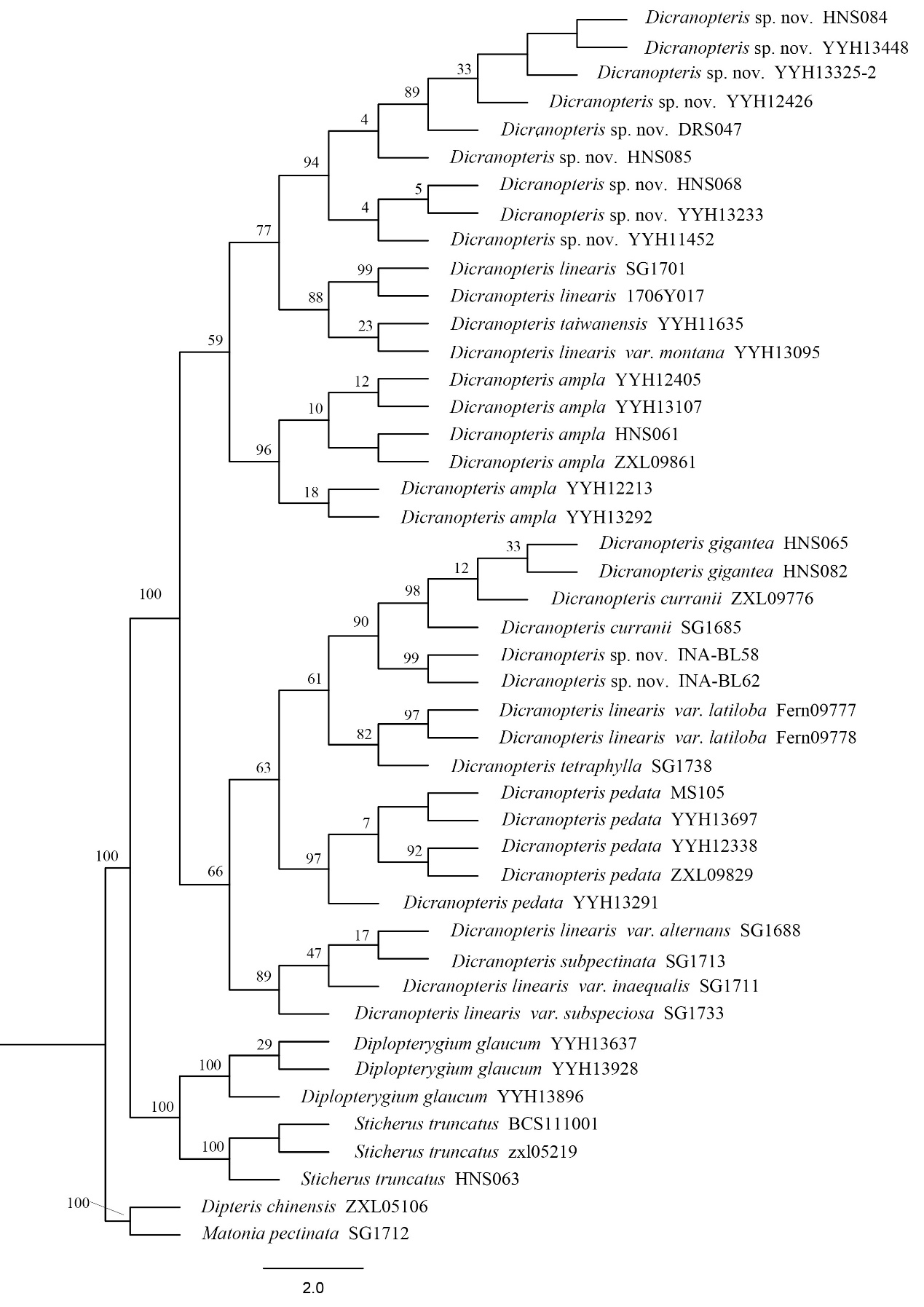


**Supplementary Figure 2.** The phylogenetic topology of *Dicranopteris* based on the concatenated dataset including sequences of *rbcL*, *atpB*, *rps4*, *trnL-trnF* and *matK* from maximum parsimony analysis.

## Supplementary Tables

**Supplementary Table 1.** Summary of the mainly previous classification treatments of *Dicranopteris*.

| **Underwood (1907)** | **Nakai (1950)** | **Holttum (1959)** | **Qin (1959)** | **Jin et al. (2013)** |
| --- | --- | --- | --- | --- |
| *D. Bancroftii* (Hook.) | *D. crassifolia* (Presl)  Nakai, comb. nova | *D. curranii* Copel. | *D. ampla*  Ching et Chiu | *D. ampla*  Ching et Chiu |
| *D. bicolor* (Christ) | *D. dichotoma*  (Thunberg) Bern. | *D. clemensiae* Holtt. | *D. dichotoma*  (Thunb.) Bernh. | *D. gigantea*  Ching |
| *D. Brunei* (Christ) | *D. discolor* (Schrader)  Nakai, comb. nova. | *D. pubigera*  (Bl.) Nakai | *D. gigantea* Ching | *D. pedata*  (Holtt.) Nakai |
| *D. costaricensis* sp. nov. | *D. divaricata* (Moore)  Nakai, comb. nova | *D. speciosa* (Presl) Holtt. | *D. linearis*  (Burm.) Underw. | *D. splendida*  (Hand.-Mazz.) Ching |
| *D. cubensis* sp. nov. | *D. ferruginea*  (Desvaux) Copel. | *D. linearis* (Burm. f.) Underw. | *D. splendida*  (Hand.-Mazz.) Ching | *D. taiwanensis*  Ching et Chiu |
| *D. farinosa* (Kaulf.) | *D.* *flexuosa*  (Schrad.) Under. | *D. linearis var. linearis* | *D. taiwanensis*  Ching et Chiu |  |
| *D.* *flexuosa* (Schrad.) | *D.* *grandis* (Fee)  Nakai, comb. nova | *D. linearis var. ferruginea*  (Bl.) Holtt. |  |  |
| *D. fulva* (Desv.) | *D. hawaiicola*  Nakai, nom. novum. | *D. linearis var. subferruginea* (Hieron) Nakai |  |  |
| *D. furcata* (L.) | *D. Hermanni* (R. Brown) Nakai, comb. nova. | *D. linearis var. rigida*  (Bl.) Holtt. |  |  |
| *D. intermedia* (Baker) | *D. Klotschii* (Hook.) Nakai, comb. nova | *D. linearis var. latiloba* Holtt. |  |  |
| *D. jamaicensis* sp. nov. | *D. Lessonii* (A. Richard) Nakai, comb. nova | *D. linearis var. subpectinata*  (Christ) Holtt. |  |  |
| *D. mellifera* (Christ) | *D. linearis* (Burm. fil.) Uderw. | *D. linearis var. subspeciosa* Holtt*.* |  |  |
| *D. orthoclada* (Christ) | *D. nervosa* Maxon | *D. linearis var. inaequalis* (Rosenst.) Holtt. |  |  |
| *D. palmata* (Schaffner) sp. nov. | *D. nitida* (Presl)  Nakai, comb. nova | *D. linearis var. alternans* (Mett.) Holtt. |  |  |
| *D. pectinata* (Willd.) | *D. opposita* (v. A. v. R.)  Nakai, comb. nova | *D. linearis var. demota* Holtt. |  |  |
| *D. pteridella* (Christ) | *D. pubigera* (Bl.)  Nakai, comb. nova | *D. linearis var. tetraphylla* (Rosenst.) Nakai |  |  |
| *D. retroflexa* (Bommer) | *D. pumila* (Martius)  Nakai, comb. nova | *D. linearis var. montana* Holtt. |  |  |
| *D. strictissima* (Christ) | *D. rigida* (Kunze)  Nakai, comb. nova | *D. linearis var. altissima* Holtt. |  |  |
|  | *D. Sartonii* (Fee)  Nakai, comb. nova |  |  |  |
|  | *D. scalpturata* (Fee)  Nakai, comb. nova |  |  |  |
|  | *D. seminuda* (Klotsch)  Nakai, comb. nova |  |  |  |
|  | *D. splendida*  (Hand.-Mazz.) Ching |  |  |  |
|  | *D. Warburgii* (Christ)  Nakai, comb. nova |  |  |  |

**Supplementary Table 2.** Information of samples for phylogenetic analysis. Dash (-) indicates unavailable data.

| **Voucher** | **Species** | **Collection** | **GenBank Accession Number** | | | | |
| --- | --- | --- | --- | --- | --- | --- | --- |
| **number** |  | **site** | ***rbcL*** | ***atpB*** | ***matK*** | ***rps4*** | ***trnL-F*** |
| DRS047 | *Dicranopteris austrosinensis* | Guangxi, China | MZ365072 | MZ365111 | MZ365139 | MZ365168 | MZ365202 |
| YYH13233 | *D. austrosinensis* | Guangxi, China | MZ365079 | MZ365100 | MZ365144 | MZ365175 | MZ365209 |
| YYH13325-2 | *D. austrosinensis* | Guangxi, Chian | MZ365075 | MZ365103 | MZ365142 | MZ365171 | MZ365205 |
| YYH13448 | *D. austrosinensis* | Guangxi, China | KU936634 | KU877800 | KU936569 | KU936699 | KU936764 |
| HNS068 | *D. austrosinensis* | Hainan, China | MZ365076 | MZ365117 | — | MZ365172 | MZ365206 |
| HNS085 | *D. austrosinensis* | Hainan, China | MZ365077 | MZ365120 | — | MZ365173 | MZ365207 |
| YYH11452 | *D. austrosinensis* | Hainan, China | MZ365078 | MZ365092 | MZ365143 | MZ365174 | MZ365208 |
| HNS084 | *D. austrosinensis* | Hainan, China | MZ365073 | MZ365119 | MZ365140 | MZ365169 | MZ365203 |
| YYH12426 | *D. austrosinensis* | Guangdong, China | MZ365074 | MZ365097 | MZ365141 | MZ365170 | MZ365204 |
| HNS061 | *Dicranopteris ampla* | Hainan, China | MZ365060 | MZ365114 | MZ365131 | MZ365156 | MZ365196 |
| YYH12213 | *D. ampla* | Yunnan, China | MZ365061 | MZ365094 | MZ365132 | MZ365157 | MZ365197 |
| YYH12405 | *D. ampla* | Guangdong, China | MZ365062 | MZ365096 | MZ365133 | MZ365158 | MZ365198 |
| YYH13107 | *D. ampla* | Xizang, China | MZ365063 | MZ365099 | MZ365134 | MZ365159 | MZ365199 |
| YYH13292 | *D. ampla* | Guangxi, China | MZ365064 | MZ365102 | MZ365135 | MZ365160 | MZ365200 |
| ZXL09861 | *D. ampla* | Thailand | MZ365065 | MZ365108 | MZ365136 | MZ365161 | MZ365201 |
| HNS065 | *Dicranopteris crunii* | Hainan, China | MZ365070 | MZ365116 | MZ365153 | MZ365166 | MZ365217 |
| HNS082 | *D. crunii* | Hainan, China | MZ365071 | MZ365118 | — | MZ365167 | MZ365218 |
| SG1685 | *D. crunii* | Malaysia | MZ365068 | MZ365124 | — | MZ365165 | MZ365215 |
| ZXL09776 | *D. crunii* | Thailand | MZ365069 | MZ365106 | MZ365152 | MZ365164 | MZ365216 |
| INA-BL58 | *Dicranopteris baliensis* | Bali, Indonesia | MZ365066 | MZ365121 | MZ365150 | MZ365162 | MZ365213 |
| INA-BL62 | *D. baliensis* | Bali, Indonesia | MZ365067 | MZ365122 | MZ365151 | MZ365163 | MZ365214 |
| YYH12338 | *Dicranopteris pedata* | Yunnan, China | MZ365081 | MZ365095 | MZ365146 | MZ365177 | MZ365220 |
| ZXL09829 | *D. pedata* | Thailand | MZ365084 | MZ365107 | MZ365149 | MZ365180 | MZ365223 |
| MS105 | *D. pedata* | Hunan, China | MZ365080 | MZ365123 | MZ365145 | MZ365176 | MZ365219 |
| YYH13291 | *D. pedata* | Guangdong, China | MZ365082 | MZ365101 | MZ365147 | MZ365178 | MZ365221 |
| YYH13697 | *D. pedata* | Guangxi, China | MZ365083 | MZ365104 | MZ365148 | MZ365179 | MZ365222 |
| SG1701 | *Dicranopteris linearis* | Malaysia | — | MZ365126 | — | MZ365190 | MZ365228 |
| 1706Y017 | *Dicranopteris linearis* | Xizang, China | MZ365087 | MZ365109 | — | MZ365183 | MZ365212 |
| YYH11635 | *Dicranopteris taiwanensis* | Taiwan, China | MZ365085 | MZ365093 | MZ365137 | MZ365181 | MZ365211 |
| YYH13095 | *D. taiwanensis* | Xizang, China | MZ365086 | MZ365098 | MZ365138 | MZ365182 | MZ365210 |
| Fern09777 | *Dicranopteris latiloba* | Indonesia | MZ365090 | MZ365112 | — | MZ365188 | MZ365227 |
| Fern09778 | *D. latiloba* | Indonesia | — | MZ365123 | — | MZ365189 | — |
| SG1688 | *Dicranopteris alternans* | Malaysia | MZ365088 | MZ365125 | — | MZ365193 | — |
| SG1733 | *Dicranopteris subspeciosa* | Malaysia | MZ365089 | MZ365130 | — | MZ365195 | MZ365231 |
| SG1713 | *Dicranopteris subpectinata* | Malaysia | — | MZ365129 | — | MZ365192 | MZ365230 |
| SG1711 | *Dicranopteris inaequailis* | Malaysia | — | MZ365127 | — | MZ365191 | MZ365229 |
| SG1738 | *Dicranopteris tetraphylla* | Malaysia | — | MZ365091 | — | MZ365194 | — |
| ZXL05219 | *Sticherus truncatus* | Guangdong, China | KU936635 | KU877801 | KU936570 | KU936700 | KU936765 |
| BCS111001 | *S. truncatus* | Bali, Indonesia | MZ365058 | MZ365110 | MZ365154 | MZ365184 | MZ365224 |
| HNS063 | *S. truncatus* | Hainan, China | MZ365059 | MZ365115 | MZ365155 | MZ365185 | MZ365225 |
| YYH13637 | *Diplopterygium glaucum* | Guangxi, China | KU936579 | KU877745 | KU936514 | KU936644 | KU936709 |
| YYH13896 | *D. glaucum* | Sichuan, China | KU936580 | KU877746 | KU936515 | KU936645 | KU936710 |
| YYH13928 | *D. glaucum* | Sichuan, China | KU936581 | KU877747 | KU936516 | KU936646 | KU877711 |
| ZXL05106 | *Dipteris chinensis* | Thailand | MZ365057 | MZ365105 | — | MZ365186 | MZ365226 |
| SG1712 | *Matonia pectinata* | Malaysia | MZ365056 | MZ365128 | — | MZ365187 | — |

**Supplementary Table 3.** Substitution models for each gene partition used in the study.

| Criterion | Partition | Codon position | MrBayes | IQ-Tree |
| --- | --- | --- | --- | --- |
| AICc | *rbcL* | 1st | K80 + I | GTR+I+G |
| AICc | *rbcL* | 2nd | HKY + I | GTR+I+G |
| AICc | *rbcL* | 3rd | GTR + G | GTR + G |
| AICc | *atpB* | 1st | GTR + I | GTR+I+G |
| AICc | *atpB* | 2nd | GTR + G | GTR + G |
| AICc | *atpB* | 3rd | GTR + I | GTR + G |
| AICc | *matK* | 1st | GTR + G | GTR + G |
| AICc | *matK* | 2nd | GTR + G | GTR + G |
| AICc | *matK* | 3rd | GTR + I | GTR + G |
| AICc | *rps4* | — | GTR + I | GTR + G |
| AICc | *trnL-F* | — | GTR + G | GTR + G |
